# Orthopædic Surgery

**Published:** 1898-10

**Authors:** 


ORTHOPÆDIC SURGERY.—By James E. Moore, M. D., Professor of Orthopædic and Clinical Surgery in the College of Medicine of the University of Minnesota, etc. Illustrated. Published by W. B. Saunders, Philadelphia, 1898. Pages, 352. Price, cloth, $2.50.

This is a text book for students and practitioners, but as the author has excluded all methods of diagnosis and treatment, except such as he has found of value, it is essentially clinical, though quite complete. Orthopædia is essentially an American science, and rich contributions have been made to it from the rich storehouse of American inventive genius. In these days of national spirit, not to say national exultation, our profession should not fail to claim its own, lest haply we shall be considered as having nothing to claim.

The subject is considered in twenty-four chapters conveniently divided and indexed, making access to its contents easy. Much attention has been given to the early diagnosis of deformities, and if this is done simple treatment and appliances are wonderfully successful. The work fairly represents the present status of the science, and shows over the complicated methods and appliances, formerly in use. J.

